# The presence of CLL-associated stereotypic B cell receptors in the normal BCR repertoire from healthy individuals increases with age

**DOI:** 10.1186/s12979-019-0163-x

**Published:** 2019-08-28

**Authors:** Alice F. Muggen, Madelon de Jong, Ingrid L. M. Wolvers-Tettero, Martine J. Kallemeijn, Cristina Teodósio, Nikos Darzentas, Ralph Stadhouders, Hanna IJspeert, Mirjam van der Burg, Wilfred FJ van IJcken, Jan A. N. Verhaar, Wayel H. Abdulahad, Elisabeth Brouwer, Annemieke M. H. Boots, Rudi W. Hendriks, Jacques J. M. van Dongen, Anton W. Langerak

**Affiliations:** 1 Department Immunology, Laboratory Medical Immunology, Erasmus MC, Dr. Molewaterplein 40, 3015 GD Rotterdam, The Netherlands; 2 Present Address: Department Immunohematology and Blood Transfusion, Leiden University Medical Center, Leiden, The Netherlands; 3 Central European Institute of Technology, Masaryk University, Brno, Czech Republic; 4 Department Internal Medicine, University Schleswig-Holstein, Kiel, Germany; 5 Department Pulmonary Medicine, Erasmus MC, Rotterdam, The Netherlands; 6 Present Address: Department Pediatrics, Leiden University Medical Center, Leiden, The Netherlands; 7 Biomics Core Facility, Erasmus MC, Rotterdam, The Netherlands; 8 Department Orthopedics, Erasmus MC, Rotterdam, The Netherlands; 9 Department Rheumatology and Clinical Immunology, University Medical Center Groningen, Groningen, The Netherlands

**Keywords:** Aging, B-lymphocyte, BCR repertoire, CLL, Stereotypic BCR

## Abstract

**Background:**

Aging is known to induce immunosenescence, resulting in alterations in both the innate and adaptive immune system. Here we evaluated the effects of aging on B cell subsets in peripheral blood of 155 immunologically healthy individuals in four age categories (range 20-95y) via multi-parameter flow cytometry. Furthermore, we studied the naive and antigen-experienced B cell receptor (BCR) repertoire of different age groups and compared it to the clonal BCR repertoire of chronic lymphocytic leukemia (CLL), a disease typically presenting in elderly individuals.

**Results:**

Total numbers and relative frequencies of B cells were found to decline upon aging, with reductions in transitional B cells, memory cell types, and plasma blasts in the 70 + y group. The BCR repertoire of naive mature B cells and antigen-experienced B cells did not clearly alter until age 70y. Clear changes in IGHV gene usage were observed in naive mature B cells of 70 + y individuals, with a transitional pattern in the 50-70y group. IGHV gene usage of naive mature B cells of the 50-70y, but not the 70 + y, age group resembled that of both younger (50-70y) and older (70 + y) CLL patients. Additionally, CLL-associated stereotypic BCR were found as part of the healthy control BCR repertoire, with an age-associated increase in frequency of several stereotypic BCR (particularly subsets #2 and #5).

**Conclusion:**

Composition of the peripheral B cell compartment changes with ageing, with clear reductions in non-switched and CD27 + IgG+ switched memory B cells and plasma blasts in especially the 70 + y group. The BCR repertoire is relatively stable until 70y, whereafter differences in IGHV gene usage are seen. Upon ageing, an increasing trend in the occurrence of particular CLL-associated stereotypic BCR is observed.

**Electronic supplementary material:**

The online version of this article (10.1186/s12979-019-0163-x) contains supplementary material, which is available to authorized users.

## Background

Changes in the immune system related to aging generally lead to increased susceptibility to infections, poor responses to new and evolving pathogens, poor vaccination responses, and higher incidence of autoimmune disorders and malignancies [1, 2]. This decline in function of the immune system, also referred to as immunosenescence, is the result of alterations occurring in both innate and adaptive immunity [3].

Age-related changes in humoral immune responses have generally been ascribed to defects in the T cell compartment and a lack of T cell help for B cell function [3]. Nevertheless, mouse studies do provide evidence for changes in the B cell compartment itself during aging. Although total B cell numbers did not alter much, shifts in the distribution of functional subsets were apparent with old age. In fact, in old mice nearly 100% of splenic B cells exhibited an antigen-experienced phenotype [4] and circulating immunoglobulins (Ig) were predominantly derived from post-germinal center B cells, as deduced from the presence of somatic hyper mutations (SHM) [5].

In human, age-related alterations in peripheral blood (PB) B cell subset distribution have also been reported, with circulating CD19+ B cells declining in absolute numbers and frequencies [6–10]. In some studies numbers and percentages of CD27+ memory B cells were found to decline [7, 8], whereas others showed an increase of these cells [10–12]. Similarly, numbers and percentages of naive CD27-IgD+ B cells were found to decrease by some studies [9, 10, 12], whereas others reported an increase [7, 8]. These inconsistent results may be explained by different B cell subset definitions and/or by large inter-individual variations in the studied age groups [11, 13].

Changes in B cell subsets during aging will likely also impact on B cell receptor (BCR) repertoire diversity. Indeed, in several mouse models age-related changes in the naive BCR repertoire were reported [14]. In some elderly humans, Ig heavy chain (IGH) complementarity determining region 3 (HCDR3) spectratyping analysis of PB B cells revealed a significant loss of diversity, which was associated with poor health status and poor survival [13]. Conflicting data, however, were reported on SHM in IGHV genes of the memory B cell compartment upon aging, varying from increased mutation rates in IgG+ but not in IgM+ PB memory B cells, to increased mutation rates in IgM+ memory B cells but not in other tonsillar subsets [15, 16].

Introduction of next generation sequencing (NGS) technologies has opened new possibilities to analyze the aging BCR repertoire, particularly in the light of immune diseases that typically arise in elderly. One category of immune diseases with a higher change to develop in elderly humans are B cell malignancies, with chronic lymphocytic leukemia (CLL) being the most common type. Notably, in about one-third of CLL patients, quasi-identical (stereotypic) BCRs are observed, which are characterized by restricted IGHV, IGHD, and IGHJ gene usage plus similarities in HCDR3 length and amino acid sequence [17]. One study reported on stereotypic BCRs within the normal IGHV1–69-IGHJ6 repertoire [18]. Little is known however about the overall existence of CLL-associated stereotypic BCRs in the normal BCR repertoire of different age groups. We hypothesize that these CLL-associated stereotypic BCR could be a reflection of changes in B cell subset distribution and the normal BCR repertoire upon aging.

In this study we determined absolute numbers and relative distribution of PB B cell subsets in healthy individuals of different age categories. Additionally, we used NGS to investigate the BCR repertoire of naive mature B cells and different types of antigen-experienced B cells in healthy individuals upon aging. Finally, we compared IGHV gene usage of the normal BCR repertoire of different age groups with that of CLL leukemic cells and evaluated the occurrence of CLL-associated stereotypic BCR in the aging normal BCR repertoire.

## Results

### Alterations in peripheral blood B cell subpopulations are minor upon aging

To study B cell dynamics during aging, we performed extensive flow cytometric immunophenotyping of peripheral blood (PB) cells in a cohort of 155 immunologically healthy individuals of 20–95 years [< 50 (*n* = 47), 50–60 (*n* = 31), 60–70 (*n* = 45), and 70+ (*n* = 32)].

**Fig. 1:**
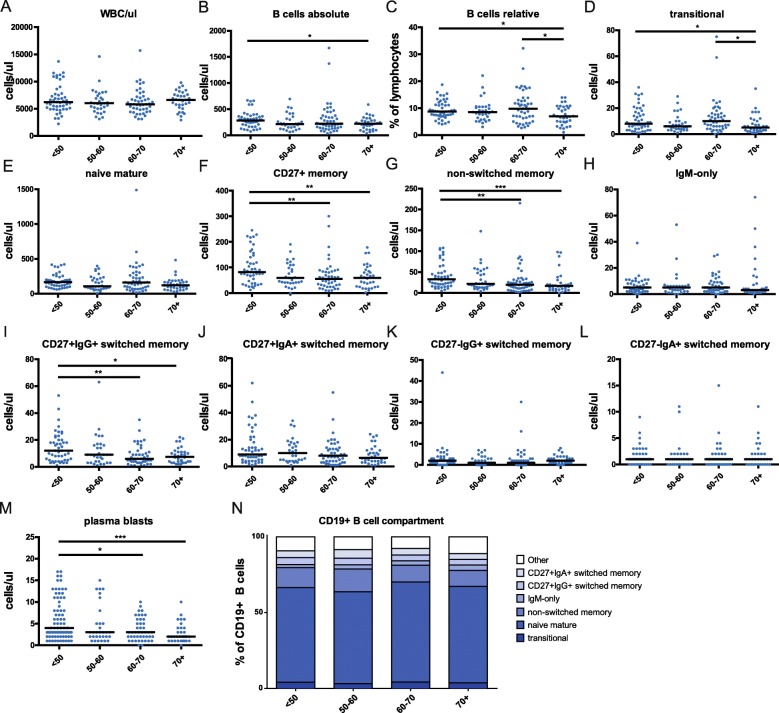
B cells and B cell subpopulations in peripheral blood upon aging. **a**. WBC counts. **b**-**c**. Analysis of total cell numbers (**b**) and relative frequencies (**c**) of B cells. **d**-**n**. Total numbers of different B cell subpopulations (**d**-**m**), and relative distribution of B cell subpopulations (**n**). Data were evaluated for four age categories: < 50 (*n* = 47), 50–60 (*n* = 31), 60–70 (*n* = 45), and > 70 (*n* = 32). Statistical significance between age groups was determined using the Mann-Whitney U test; *, *p* < 0.05; **, *p* < 0.01; ***, *p* < 0.001; ****, *p* < 0.0001

To validate our cohort, we first evaluated age-related dynamics of the total white blood cells and lymphocyte subpopulations (Fig. 1**;** Additional file 1: Figure 1). White blood counts (WBC) remained stable across the age groups (Fig. 1A). Although differences were not significant, there was a trend that the absolute numbers of lymphocytes was lower in the 50-60y and 60-70y groups than in the <50y group (Additional file 1: Figure S1). Both absolute and relative numbers of naive CD8+ T cells significantly declined >50y of age, whereas absolute and relative numbers of CD8+ effector (TemRA) T cells clearly increased with increasing age and CD8+ effector memory (TemRO) T cells remained stable (data not shown). CD4+ T cell and NK cell numbers did not alter between the age groups (Additional file 1: Figure S1). These data are in line with previously reported data on T and NK cells [19], thus supporting the validity of our cohort for evaluating B cell aging effects.

Next, we focused on the composition of the B cell compartment (see Methods, Additional file 6: Table S1, Additional file 2: Figure S2) in the different age groups. The total B cell numbers and relative frequencies of B cells (as fraction of total lymphocytes) slightly declined during aging, resulting in a significant difference between the <50y and 70 + y groups (Fig. 1B-C). More specifically, we observed a significant reduction in absolute numbers of transitional B cells in the 70 + y group, as well as reductions in the non-switched and CD27 + IgG+ switched memory B cell populations, and plasma blasts in the two oldest age categories (Fig. 1D-M). For naive mature B cells and all other types of memory B cells no clear alterations in absolute numbers were noted upon aging (Fig. 1D-M). The overall PB B cell subset distribution displayed only minor shifts between different age groups (Fig. 1N), which was mainly reflected by the significantly lower frequencies of non-switched memory B cells and plasma blasts upon increasing age (Additional file 3: Figure S3).

**Fig. 2:**
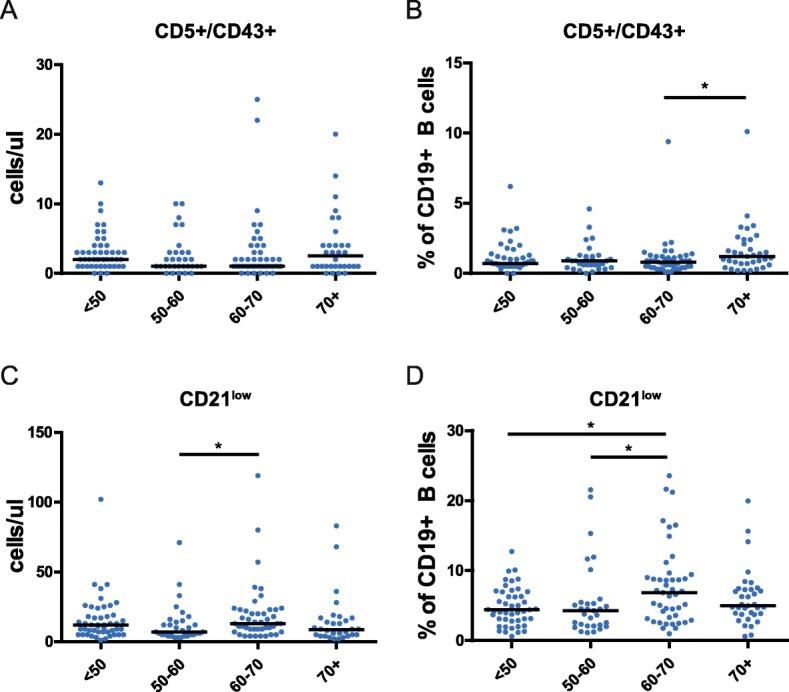
CD43 + CD5+ B cells and CD21^low^ B cells in peripheral blood upon aging. Analysis of total cell numbers (**a**,**c**) and relative frequencies (**b**,**d**) of CD5 + CD43+ B cells and CD21^low^ B cells, respectively. Data were evaluated for four age categories: < 50 (n = 47), 50–60 (n = 31), 60–70 (n = 45), and > 70 (n = 32). Statistical significance between age groups was determined using the Mann-Whitney U test; *, p < 0.05; **, p < 0.01; ***, p < 0.001; ****, p < 0.0001

We then looked into CD5 + CD43+ chronically activated B cells, as these have been associated with CLL and CLL-like MBL that typically appear in elderly [20–22]. We noted a small but significant increase in relative frequencies of CD5 + CD43+ B cells in the 70 + y group (when compared with the 60-70y age group), together with a trend towards increased absolute numbers of these cells (Fig. 2A-B). Next, we also evaluated CD21^low^ B cells, as high numbers of these cells have been associated with autoimmune disease [23]. Notably, a significant increase of CD21^low^ cell numbers and relative frequencies was seen between the 50-60y and 60-70y groups, which normalized again in the 70 + y group (Figs. 2C-D).

Taken together, our B cell subpopulation analysis mostly showed a decline in transitional B cells, non-switched and CD27 + IgG+ switched memory B cells, and plasma blasts in elderly. The frequency of CD21^low^ B cells appeared to be increased in 60-70y group.

### Composition of the BCR repertoire of naïve mature B cells is stable until 70y but shows changes in the 70 + y group

**Fig. 3:**
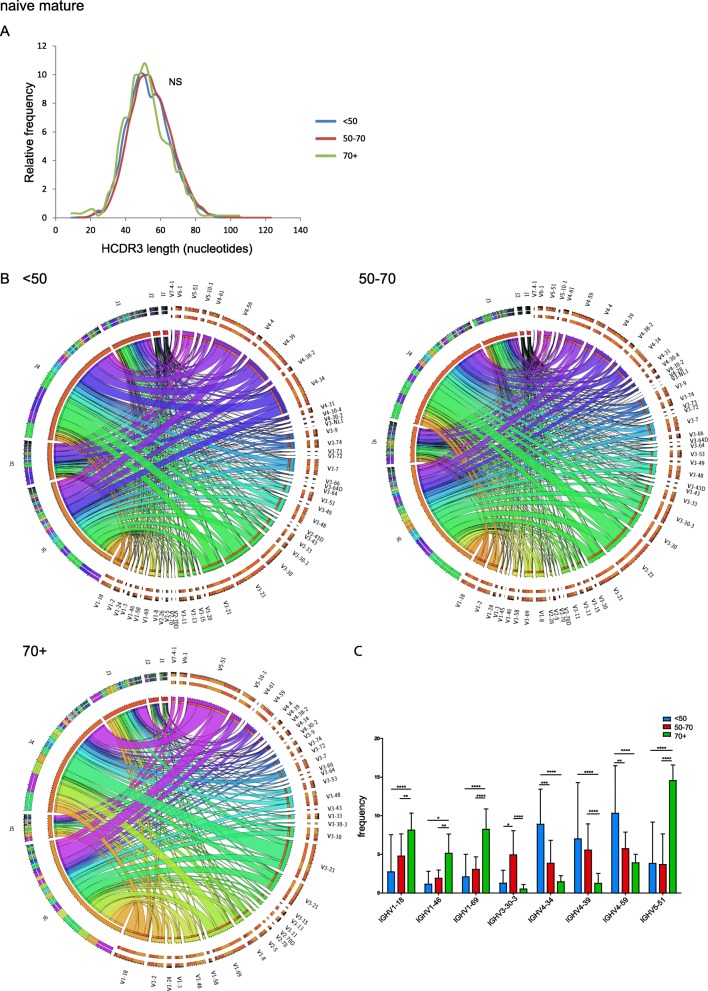
BCR repertoire analysis in naive mature B cells upon aging. **a**. Comparison of HCDR3 lengths in < 50, 50–70, and 70 + y age categories. Statistical significance was determined using the Kolmogorov-Smirnov test. **b**. Circos plots of the combination of IGHV-IGHJ gene usage in < 50, 50–70, and 70 + y age categories. **c**. Differences in IGHV gene usage frequencies upon aging. Statistical significance between different age groups was determined using the two-way ANOVA test (with Bonferroni multiple comparisons correction); *, *p* < 0.05; **, *p* < 0.01; ***, *p* < 0.001; ****, *p* < 0.0001

Our next aim was to see which differences occur in the BCR repertoire of healthy donors during aging. To this end, we first sorted antigen-inexperienced naive mature B cells of healthy controls in the <50y, 50-70y, and 70 + y groups (*n* = 4–5 per age group) and analyzed unique IGHV-IGHD-IGHJ sequences and their HCDR3 regions (Additional file 6: Table S2). Despite slight variations in the mean HCDR3 lengths between the <50y (53.0 nucleotides), 50-70y (54.5 nucleotides), and 70 + y (46.1 nucleotides) groups, the overall HCDR3 profiles showed no significant differences (Fig. 3A).

When evaluating gene usage, differences in IGHV subgroup usage between the <50y and 50-70y groups appeared limited, but we did find a marked increase in IGHV1 and IGHV5 subgroup usage and a decrease in IGHV4 subgroup usage in the 70 + y donors (Fig. 3B). Upon further examination of IGHV gene usage, significant differences were mostly noted in the 70 + y age group, with IGHV1–18, IGHV1–46, IGHV1–69, and IGHV5–51 gene usage being significantly higher, and IGHV4–34, IGHV4–39, and IGHV4–59 usage being significantly lower (Fig. 3C), which could not be explained by small clonal proliferations. That said, the most commonly used IGHV gene in all three age groups appeared to be the IGHV3–23 gene, followed by IGHV3–21 (Fig. 3B). We did not detect clear differences in IGHD and IGHJ gene usage between any of the three age groups.

Collectively, our data from healthy controls of different age groups suggest that the BCR repertoire of naive mature B cells is relatively stable until 70y. In contrast, in the 70 + y group IGHV gene usage does differ, which most probably should be interpreted as an aging effect, since there were no other indications that can explain this difference.

### Differences in BCR repertoire of memory B cell subpopulations are minor between <50y and 50-70y age groups

To evaluate age effects in the antigen-selected BCR repertoire, we then focused on non-switched, IgM-only, and CD27 + IgG+ switched memory B cells, which are all antigen-experienced cells though arising via distinct activation routes. Some of the memory B cell populations are so small that they can only be sorted from buffy coats of healthy donors (*n* = 4–5 per age group); unfortunately blood donors are only allowed to give blood until 70y, so we could not the study the 70 + y age group. Mean HCDR3 lengths did not differ significantly between the <50y and 50-70y age groups (Additional file 4: Figure S4). IGHV, IGHD, and IGHJ gene usage and combined IGHV / IGHJ patterns of non-switched, IgM-only, and CD27 + IgG+ memory B cells did not show significant differences either between these two age groups (Additional file 5: Figure S5). Notably, when analyzing SHM frequencies for these memory B cell subpopulations, we did detect a higher mutation rate for non-switched and IgM-only memory B cells in the 50-70y group, whilst a small reduction in mutation frequency was seen in CD27 + IgG+ switched memory B cells (Additional file 5: Figure S5).

Collectively these data show that the BCR characteristics of memory B cell subpopulations do not differ statistically between the <50y and 50-70y groups.

### IGHV gene usage in the clonal BCR repertoire of CLL patients is largely comparable to IGHV genes in naive mature B cells of 50-70y individuals

**Fig. 4:**
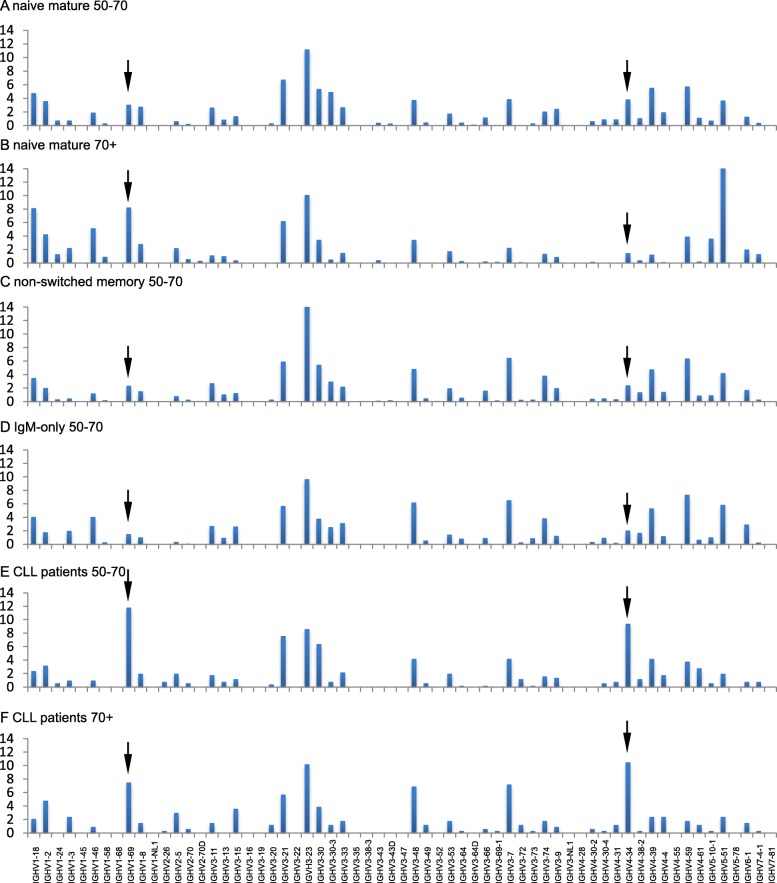
Comparison of IGHV gene usage in the normal BCR repertoire of healthy individuals and the clonal CLL repertoire of different age groups. **a**-**b**. IGHV gene usage in naive mature B cells of 50-70y (A) and 70 + y (**b**) healthy controls. **c**-**d**. IGHV gene usage in non-switched (**c**) and IgM-only B (**d**) cells of 50-70y healthy controls. E-F. IGHV gene usage in clones of CLL patients of 50-70y (**c**) and 70 + y (**d**). Statistical analysis was determined using the Fisher’s exact test; see text for details on significance levels

As the BCR is known to play an important role in disease onset and prognosis of CLL, which normally develops at elderly age (average 70 + y), we then asked whether overall IGHV gene usage in CLL patients of different age groups would reflect the BCR repertoire of normal B cells of the same age groups. To this end we evaluated IGHV gene usage in different B cell subsets of healthy controls of different age groups and compared the profiles with Sanger sequencing-based data of a cohort of 920 CLL patients (Fig. 4).

Overall IGHV gene usage profiles did not differ significantly between different types of normal B cell subpopulations, such as naive mature B cells, non-switched memory B cells, and IgM-only memory B cells, in the 50-70y age group (*p* = 0.99–1 in all comparisons). Unfortunately, no such comparison was possible in 70 + y individuals, due to the lack of available data from non-switched and IgM-only memory B cells for this age group. However, overall IGHV gene usage profiles in naive mature B cells of healthy individuals did clearly vary between the 50-70y and 70 + y groups (*p* = 0.005).

When we next compared overall IGHV gene usage profiles between CLL patients of the 50-70y and the 70 + y groups we did not observe a significant difference (*p* = 0.995). The IGHV profiles of naive mature B cells and CLLs in the 50-70y group appeared to look rather similar (*p* = 0.574), albeit with a more dominant IGHV1–69 and IGHV4–34 usage in CLL. In contrast, naive mature B cell and CLL IGHV gene usage profiles in the 70 + y groups were clearly different (*p* < 0.0001), while the overall IGHV profile in 70 + y CLL was in fact rather similar to that of naive mature B cells of the 50-70y age group (*p* = 0.110). Furthermore, overall IGHV profiles in the 50-70y group appeared clearly different between CLL and non-switched memory B cells (*p* = 0.028) or IgM-only memory B cells (*p* = 0.004).

These data indicate that the overall IGHV gene usage profile in CLL patients, irrespective of the age of presentation, is similar to naive mature B cells of especially the 50-70y control group. The overall IGHV profile of the 70 + y control group is different without obvious explanation as mentioned above.

### CLL-associated stereotypic BCR are present in naive mature B cells and increase with age

**Fig. 5:**
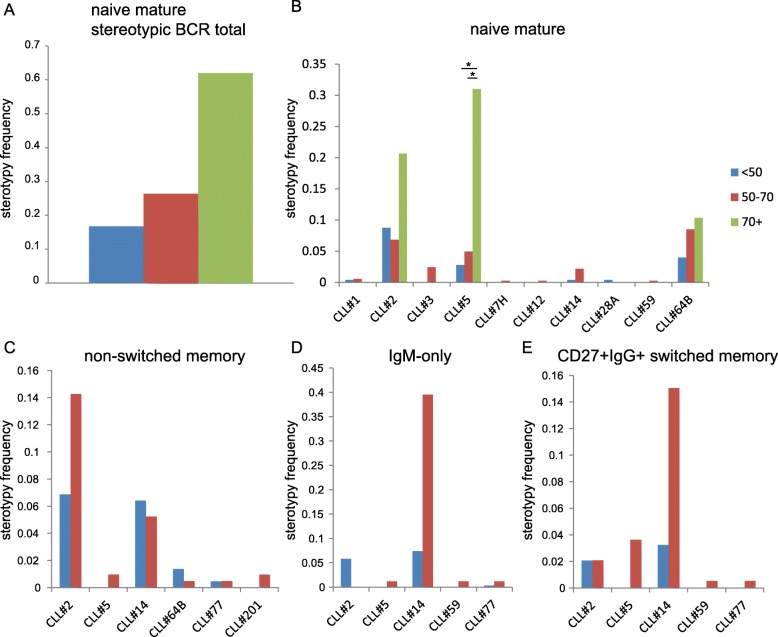
Stereotypic BCR in the normal BCR repertoire of healthy aging individuals. **a**. Total frequency of stereotypic BCR in the normal BCR repertoire in naive mature B cells of healthy individuals. **b**-**e**. Frequency of stereotypic BCR assigned to CLL-associated stereotypic subsets in naive mature (**b**), non-switched (**c**), IgM-only (**d**), and CD27 + IgG+ switched (**e**) memory B cells. Statistical significance between different age groups was determined using the two-way ANOVA test (with Bonferroni multiple comparisons correction); *, *p* < 0.05; **, *p* < 0.01; ***, *p* < 0.001; ****, *p* < 0.0001

In view of the occurrence of quasi-identical (stereotypic) BCR with similar IGHV / IGHD / IGHJ and HCDR3 features in CLL clones of different patients, we then investigated whether we could also detect stereotypic BCR in healthy individuals of different age groups. To identify CLL-related stereotypic BCRs in the normal repertoire of healthy controls of different age groups, a reference database of stereotypic BCR from CLL patients [24] was used for assigning individual IGH sequences from healthy controls. With this algorithm we could indeed identify CLL-associated stereotypic BCRs based on HCDR3 characteristics in the repertoire of naive mature B cells, and also of non-switched, IgM-only, and CD27 + IgG+ switched memory B cells (Fig. 5). The presence of stereotypic BCR receptors in naive mature B cells showed an increasing trend with age and was most apparent in the 70 + y group (Fig. 5A). In naive mature B cells, the most prominent stereotypic BCR belonged to CLL subsets #2, #5, and #64B (Fig. 5B). In non-switched memory B cells the most frequently found stereotypic BCRs concerned CLL subsets #2 and #14 (Fig. 5C). The CLL#14 BCR also appeared most prominent in IgM-only and CD27 + IgG+ switched memory B cells, especially in the 50-70y category (Fig. 5D-E).

From these findings we conclude that CLL-associated stereotypic BCRs are present in the normal BCR repertoire during life and that their frequency showed an increasing trend upon aging.

## Discussion

Here we evaluated age-related changes in B cell subset composition as well as BCR repertoire with a focus on CLL-associated stereotypic BCR usage. In the most elderly (70 + y) individuals we observed a decline in absolute numbers of transitional B cells, total memory B cells, and plasma blasts. The BCR repertoire of naive mature B cells and distinct memory B cell populations was found to be relatively stable until 70y. In naive mature B cells of the 70 + y group differences were noted, especially with respect to IGHV gene usage. Notably, IGHV gene usage in the clonal BCR repertoire in CLL did not differ with the age of presentation of the CLL patients, and largely resembled naive mature B cells of the 50-70y group rather than the 70 + y group. Finally, CLL-associated stereotypic BCR were found as part of the BCR repertoire of healthy individuals and their frequencies increased with age.

The observed decline in total B cell numbers in the 70 + y group could result from a reduced output from the bone marrow [25, 26]. An alternative explanation could be the reduction in CD27+ antigen-experienced B cell subpopulations. Non-switched and CD27 + IgG+ memory B cells together make up the majority of the CD27+ memory B cell compartment, and were previously described to decline upon aging [6]. The decline in non-switched memory B cells could underlie the higher vulnerability to diseases caused by encapsulated bacteria like *Streptococcus pneumoniae* in elderly [27]. CD27 + IgG+ memory B cells are mainly, albeit not exclusively, formed in T cell-dependent immune responses and play a role in recall responses to previously encountered pathogens [28]. The reduction of plasma blasts upon aging is in line with earlier observations [29] and fits the lower immunoglobulin levels in the circulation as reported in elderly [30]. Together these data could, at least partially, explain the reduced effects of vaccination and immune responses against infections in elderly.

Chronically activated B cells express CD5 and CD43 [31, 32] and might trigger MBL onset. [33, 34]. MBL are found in healthy adult individuals, with an incidence that increases with age to roughly 10% of individuals >65y [35]. Based on their phenotypical association with MBL [20] and CLL [22], the increase of CD5 + CD43+ B cells upon aging might thus correlate with the higher risk of MBL and CLL clones in elderly. Another B cell subset related with chronic activation concerns CD21^low^ B cells, increased numbers of which can be found in patients showing chronic inflammation in the context of autoimmune disease [23]. As we excluded individuals with inflammatory and (auto)immune disease in our immunologically healthy cohort, unfortunately we could not link the higher number of CD21^low^ B cells in the 60-70y group to overt autoimmune disease occurrence. Nevertheless, increased numbers of CD21^low^ B cells in this age group might reflect an increased incidence of, yet undiagnosed, autoimmune diseases upon aging.

BCR repertoire changes were most apparent in naive mature B cells of the 70 + y group. Since naive mature B cells are not affected by exogenous antigen, this is most likely the result of changes in repertoire development and/or output from the bone marrow. Whilst HCDR3 length, IGHD, and IGHJ usage remained stable in all three age categories, IGHV gene usage did reveal alterations. Interestingly, IGHV4–34 usage, a gene often associated with autoimmunity, was found to be reduced upon aging in these healthy individuals. Upon aging we also noticed a combined increased usage of IGHV5–51 and IGHV1–69, in line with a previous report [36]. IGHV1–69 has been associated with broadly neutralizing antibodies against amongst others influenza, HIV, hepatitis C, and commensal bacteria antigens in the context of CLL [37].

Remarkably, IGHV gene usage in both the 50-70y and 70 + y CLL patient groups most closely resembled IGHV gene usage in naive mature B cells of 50-70y, but not 70 + y, healthy individuals. One could speculate that CLL clones, even the ones in elderly CLL patients (70 + y), would have developed from B cells with a BCR repertoire of relatively younger age (<70y), but it might also reflect selection for IGHV specificities in the younger repertoire that could be predisposing for CLL development.

Stereotypic BCR, which are found in roughly one third of CLL clones, have previously been documented in RNA from a total lymphocyte pool of three healthy individuals (age 50, 69, and 69) [38, 39]. In this study we show that stereotypic BCR can be found in the normal BCR repertoire of both naive mature and antigen-experienced B cells. Interestingly, a frequently observed stereotypic BCR was the CLL#2 BCR (IGHV3–21 in combination with IGHJ6 with a short HCDR3 length of 9 amino acids), which is the most common stereotypic BCR seen in CLL patients and is associated with an aggressive form of CLL [17, 40]. Stereotypic CLL#5 BCR (IGHV1–69, IGHD3–10/3–3, IGHJ6, 20 amino acids HCDR3) as well as stereotypic CLL#64 BCR (IGHV3 subgroup genes, IGHD2 subgroup genes, IGHJ6, 21 amino acids HCDR3) [17] were relatively often detected in naive mature B cells of especially individuals 70 + y. In antigen-experienced B cells, stereotypic CLL#14 BCR (IGHV4–4, no specific IGHD, IGHJ4, and short 10 amino acids HCDR3) was frequently found. The possibility to detect stereotypic BCRs with short HCDR3 lengths in antigen-experienced B cell subpopulations would be in line with our observation that on average the complete memory BCR repertoire shows selection for shorter HCDR3 lengths in comparison with naive B cells. Notably, other common CLL-associated stereotypic BCR, such as CLL#4, CLL#6, and CLL#8 BCR, could not be detected.

Even though the frequency of stereotypic BCR in healthy B cells shows a trend towards increase with age, stereotypic BCR can already be detected in cord blood (data not shown) and thus are to be considered as part of the normal BCR repertoire. Moreover, even though the increase of CLL-associated stereotypic BCR in the aging normal BCR repertoire might imply an increased predisposition for CLL development in elderly, it should be stressed that two thirds of CLL show heterogeneous BCR specificities that can also mediate derailment of B cells leading to CLL. Investigations into the presence of CLL-associated stereotypic BCR in healthy individuals should therefore be extended to larger datasets including more healthy individuals of all age groups, as well as to individuals suffering from chronic infection, immunodeficiency, or autoimmune disease. Such studies would allow to define the true impact of CLL-associated stereotypic BCR in CLL development.

## Conclusion

We analyzed the peripheral B cell compartment and BCR repertoire during human ageing. Composition of the peripheral B cell compartment changes with ageing, with clear reductions in non-switched and CD27 + IgG+ switched memory B cells and plasma blasts in especially the 70 + y group. The BCR repertoire is relatively stable until 70y age, whereafter differences in IGHV gene usage are seen. Upon ageing, an increase in the occurrence of particular CLL-associated stereotypic BCR is observed, potentially reflecting the occurrence of such BCR in CLL in elderly.

## Methods

### Sample inclusion

For B cell subpopulation analysis, peripheral blood (PB) of immunologically healthy individuals was obtained from pre-surgery patients (Dept. Orthopedics, Erasmus MC) with the following *exclusion* criteria: (auto)immune or inflammatory diseases; malignancies; usage of anti-inflammatory or immunosuppressive drugs; surgery in the past 30 days; alcohol and drug abuse. To increase the number of subjects per age group, additional samples from the SENEX healthy aging cohort (Rheumatology and Clinical Immunology, UMCG, Groningen, Netherlands), and samples from co-workers from the department were included. Subjects (*n* = 155) were divided into four age categories: < 50 (*n* = 47), 50–60 (*n* = 31), 60–70 (*n* = 45), and 70+ (*n* = 32). For the BCR repertoire study, peripheral blood samples (*n* = 5 per age group) were additionally obtained from Sanquin Blood bank (Amsterdam, The Netherlands). Diagnostic samples from CLL patients were collected upon informed consent and anonymized for further usage. Written consent was obtained in accordance with the Declaration of Helsinki after medical ethics committee approval (MEC 2011–409, 2016–202, and 2,012,375).

### Immunophenotyping of B cell subpopulations

Folllowing white blood cell count (WBC) measurement on a Coulter®Ac.T diff analyzer (Beckman Coulter, Fullerton, CA, USA), flowcytometry was performed on whole blood [after red blood cell lysis with ammonium chloride] using an LSR Fortessa™ (BD Biosciences, San Jose, CA). Absolute cell counts of monocytes, natural killer (NK) cells, T cells, and B cells were calculated from WBC numbers using Infinicyt software (Cytognos, Salamanca, Spain). Lymphocytes were first gated based on FSC / SSC characteristics, and B cells were defined by expression of the pan-B cell marker CD19. Further gating was performed for defined CD19+ B cell subpopulations, i.e. transitional B cells (CD38hi/CD27-), naive mature B cells (CD38−/CD27−/IgM+/IgD+), non-switched memory B cells (CD38−/CD27+/IgM+/IgD+), IgM-only B cells (CD38−/CD27+/IgM+/IgD-), switched memory B cells (CD38−/CD27+ or −/IgM−/IgD-IgG+ or IgA+ or IgE+), plasma blasts (CD38^hi^/CD27+), CD5+/CD43+ B cells (CD38−/CD5+/CD43+), and CD21^low^ B cells (CD38^dim^/CD21^low^) according to published data [28] (see also (Additional file 6: Table S1), using 11-color flowcytometric stainings (Additional file 6: Table S3) and FACS DIVA software (BD Biosciences) for analysis.

### Sorting of B cell subpopulations and DNA isolation

PB mononuclear cells (PB-MNC) were isolated via Ficoll Paque gradient centrifugation. Subsequently, B cells were purified with human CD19 MicroBeads via AutoMACS (Myltenyi Biotech, Bergisch Gladbach, Germany). Next, several B cell subpopulations (naive mature, non-switched memory, IgM only, and CD27+/IgG+) were collected using a FACSAria cell sorter (BD Biosciences). Immediately after collection cells were lysed in RLT+ buffer (QIAGEN, Valencia, CA) complemented with β-mercapto-ethanol. Cells were used for DNA isolation with the DNA/RNA/miRNA Easy kit (QIAGEN) and/or stored in − 80 °C for later processing.

### NGS-based BCR repertoire analysis of healthy individuals

IGHV-IGHD-IGHJ rearrangements were amplified from 100 ng DNA of sorted B cell subpopulations (from *n* = 3–5 healthy controls per age group; see Additional file 6: Table S3) using the BIOMED-2 IGH multiplex PCR with 6 IGHV primers and 1 IGHJ consensus primers that were extended with adapter sequences for NGS. PCR products were purified by gel extraction (QIAGEN) and subsequently by Agencourt AMpure XP beads (Beckman Coulter, Brea, CA). Concentrations were measured using Quant-iT Picogreen dsDNA assay (Invitrogen, Carlsbad, CA). PCR products were sequenced on a 454 GS junior (Roche, Branford, CT), using the GS Junior Titanium emPCR, sequencing, and PicoTiterPlate kits (Roche), and partly on a MiSeq (Illumina, San Diego, CA) platform. Cross-validation experiments using B cells from healthy individuals showed comparability of data from both platforms (unpublished; see Additional file 7). Sequences were demultiplexed based on their multiplex identifier sequence and trimmed via the ImmunoGlobulin galaxy (IGGalaxy) pipeline [41]. FASTA files were uploaded in IMGT/High-V-QUEST (http://www.imgt.org/HighV-QUEST/login.action) and IMGT output files were further analyzed in the Antigen Receptor Galaxy pipeline, as described before [42].

For comparison purposes, a local Erasmus MC cohort of 920 CLL patients (mean 65y) was used, in which Sanger sequencing-based IGHV mutation status analysis was performed using the BIOMED-2 primers and protocol [43], and following ERIC interpretation guidelines [44].

IGHV-IGHJ circos plots were generated via the Circos Table Viewer (http://mkweb.bcgsc.ca/tableviewer/). CLL-associated stereotypic BCR in healthy control samples were defined using the ARResT/AssignSubsets tool (http://tools.bat.infspire.org/arrest/assignsubsets/).

### Statistical analysis

Significant differences in relative and absolute numbers of lymphocyte subpopulations and IGH SHM levels between age groups were determined using the Mann-Whitney U test. Differences in HCDR3 lengths were evaluated using Kolmogorov-Smirnov statistics. Relative IGHV gene usage and relative frequencies of stereotyped BCR between different age groups were analyzed using a two-way ANOVA with multiple comparisons. Overall IGHV gene usage between B cell subpopulations and CLL clones was analyzed using the Fisher’s exact test. A *p*-value < 0.05 was considered significant. Statistics were performed in GraphPad Prism v5.0 (La Jolla, CA).

## Additional files

Additional file 1: **Figure S1.** Frequencies and absolute numbers of T cell subsets and NK cells to validate the cohort for evaluating peripheral blood B cell subpopulations upon aging. (PDF 472 kb)

Additional file 2: **Figure S2.** Scheme of different human B-cell subpopulations in peripheral blood. (PDF 209 kb)

Additional file 3: **Figure S3.** Relative frequencies of B cell subpopulations in peripheral blood upon aging. (PDF 450 kb)

Additional file 4: **Figure S4.** No difference in HCDR3 lengths of antigen-experienced B cells upon aging. (PDF 239 kb)

Additional file 5: **Figure S5.** Minor differences in BCR repertoire of antigen-experienced B cell subpopulations in different age groups. (PDF 8560 kb)

Additional file 6: **Table S1.** Definition of human B cell subpopulations. **Table S2** Overview of productive and unique IGH sequences in NGS analysis. **Table S3** Composition of different antibody panels for staining B cell subpopulations. (DOCX 26 kb)

Additional file 7: Cross-validation of IGH assay between different NGS platforms. (DOCX 32 kb)

## Data Availability

The datasets used and/or analysed during the current study are available from the corresponding author on reasonable request.

## Abbreviations

BCR: B cell receptor
CLL: Chronic lymphocytic leukemia
IG: Immunoglobulin
IGH: Immunoglobulin heavy chain gene complex
IGHD: Immunoglobulin heavy chain diversity gene
IGHJ: Immunoglobulin heavy chain joining gene
IGHV: Immunoglobulin heavy chain variable gene
MBL: Monoclonal B cell lymphocytosis
SHM: Somatic hypermutation
