# Supplementary figures and images for: The presence of CLL-associated stereotypic B cell receptors in the normal BCR repertoire from healthy individuals increases with age
Source: Immun Ageing. 2019 Aug 28;16:22. doi: 10.1186/s12979-019-0163-x (PMC6714092; doi:10.1186/s12979-019-0163-x)

Supplementary figure 1

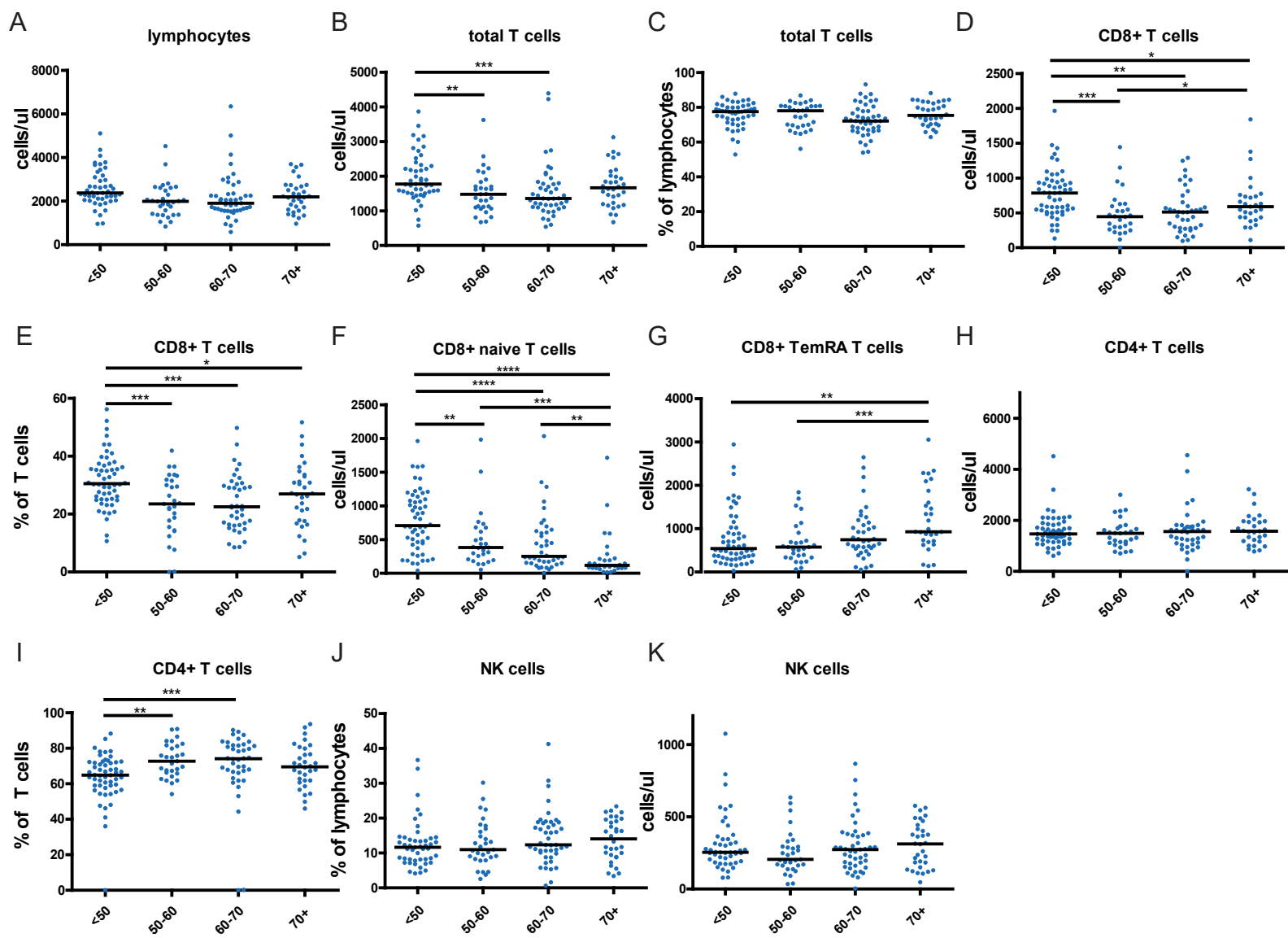

Supplement: Supplementary file 1 — Figure S1. Frequencies and absolute numbers of T cell subsets and NK cells to validate the cohort for evaluating peripheral blood B cell subpopulations upon aging. (PDF 472 kb) [file 12979_2019_163_MOESM1_ESM.pdf]

A

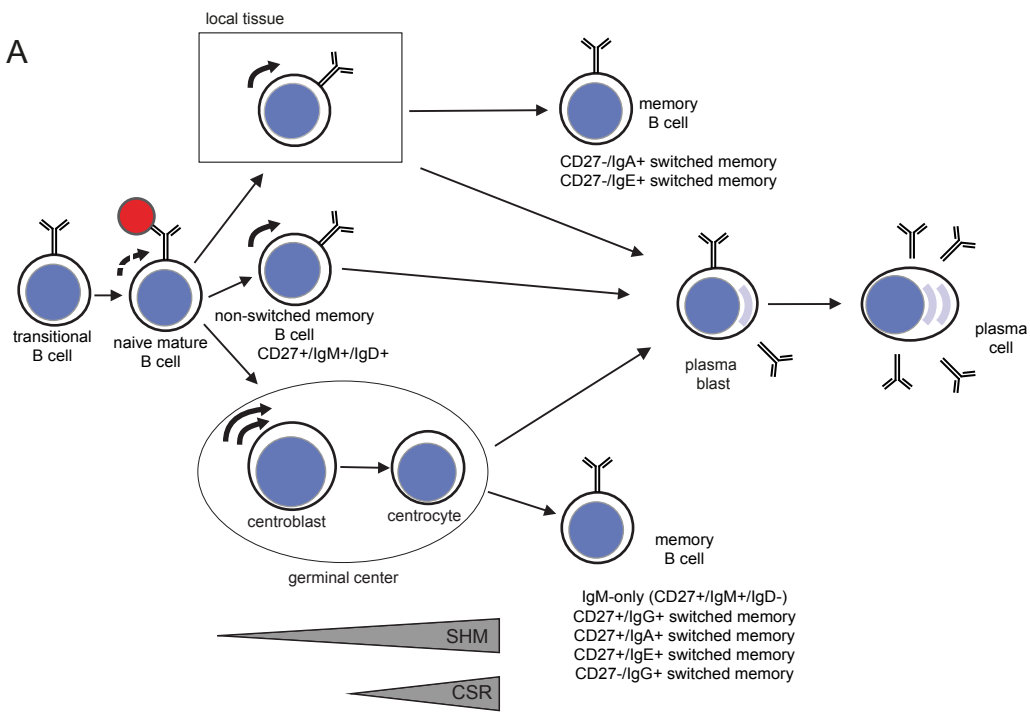

Supplement: Supplementary file 2 — Figure S2. Scheme of different human B-cell subpopulations in peripheral blood. (PDF 209 kb) [file 12979_2019_163_MOESM2_ESM.pdf]

Supplementary Figure 3

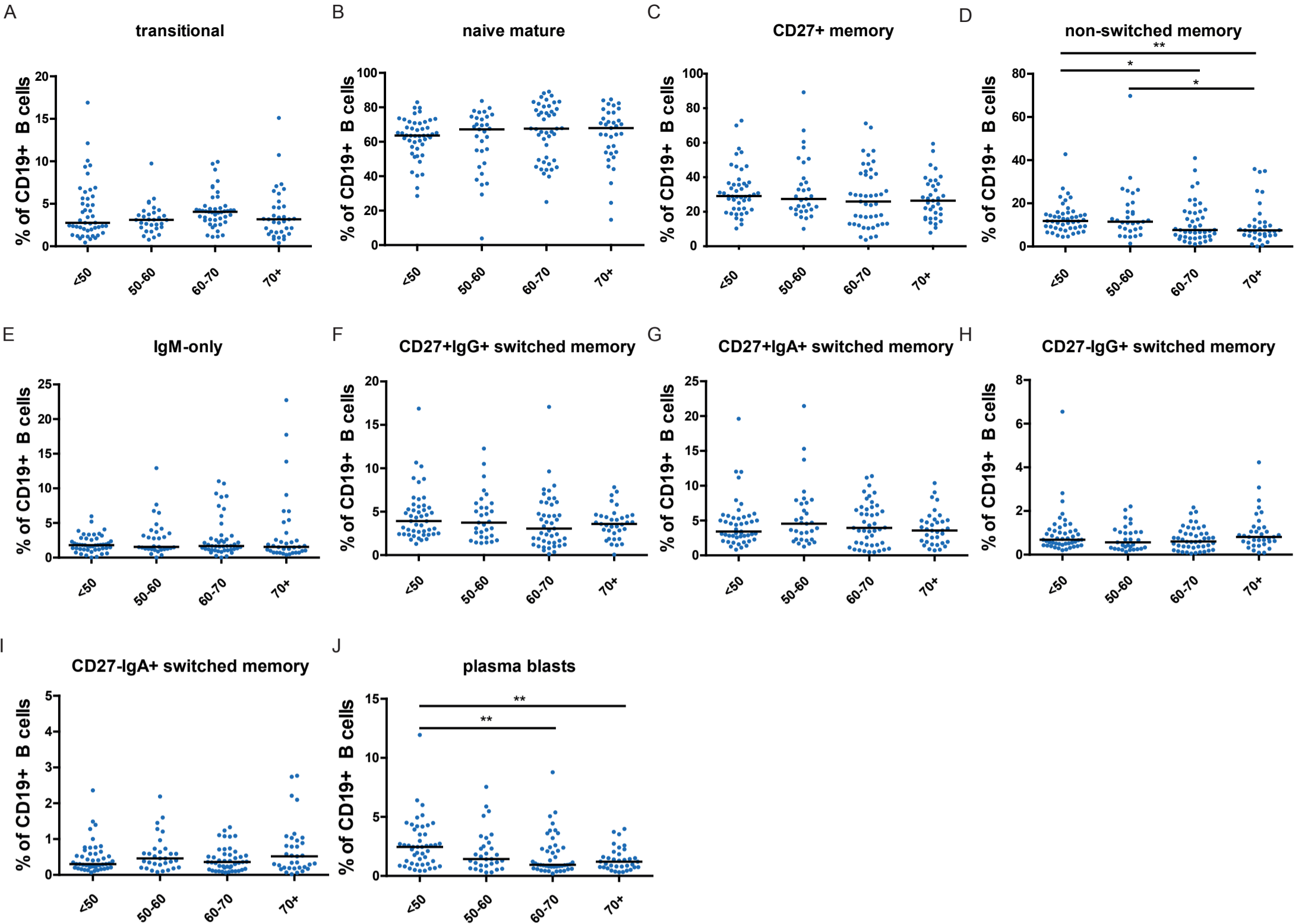

Supplement: Supplementary file 3 — Figure S3. Relative frequencies of B cell subpopulations in peripheral blood upon aging. (PDF 450 kb) [file 12979_2019_163_MOESM3_ESM.pdf]

# Supplementary Figure 4

A non-switched memory

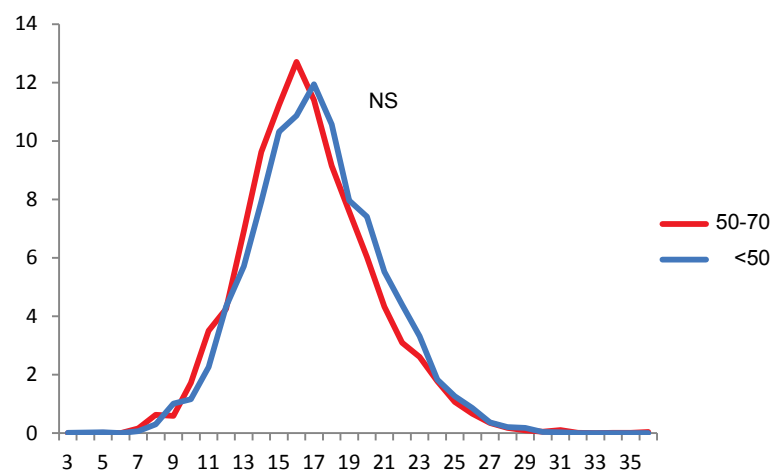

B IgM-only

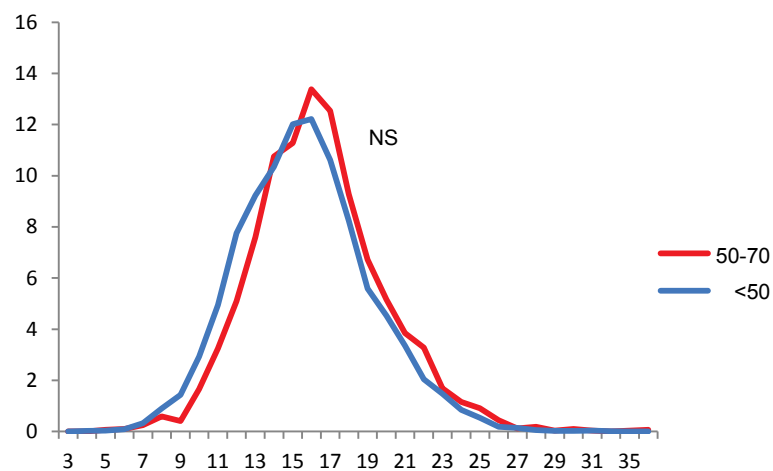

C CD27+IgG+ switched memory

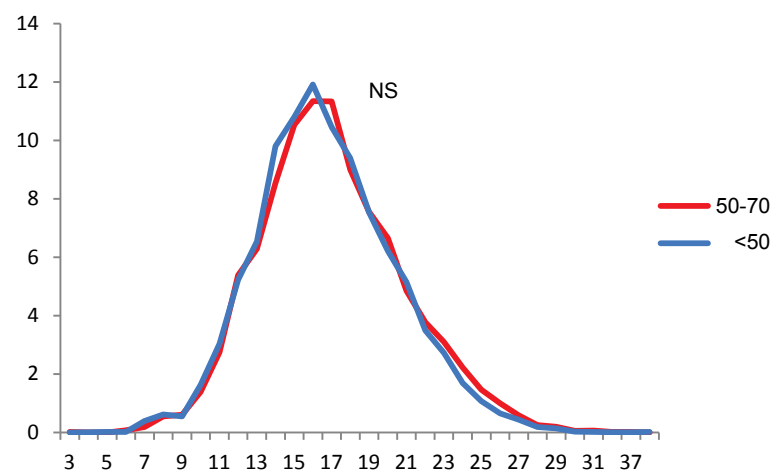

Supplement: Supplementary file 4 — Figure S4. No difference in HCDR3 lengths of antigen-experienced B cells upon aging. (PDF 239 kb) [file 12979_2019_163_MOESM4_ESM.pdf]

# Supplementary Figure 5

## A IgM-only

<50

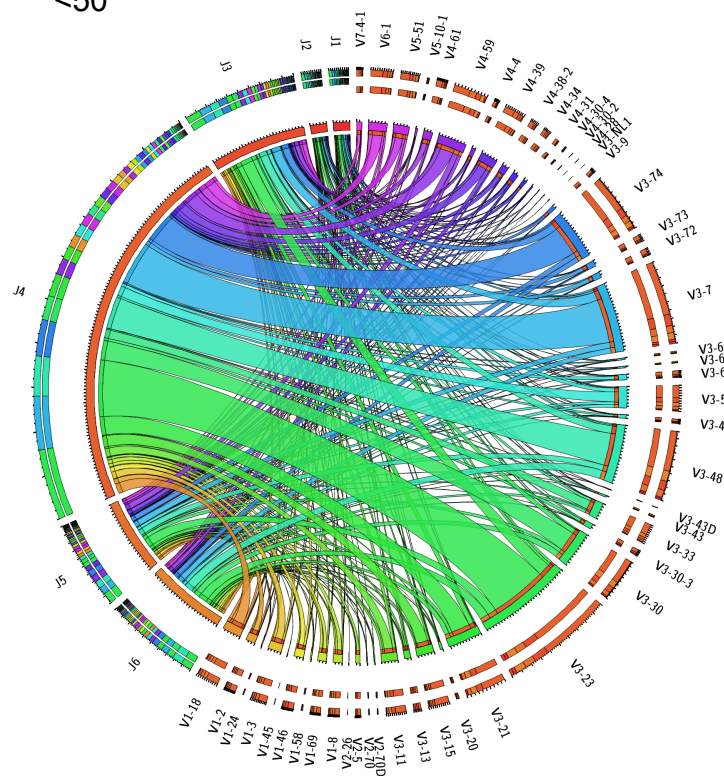

50-70

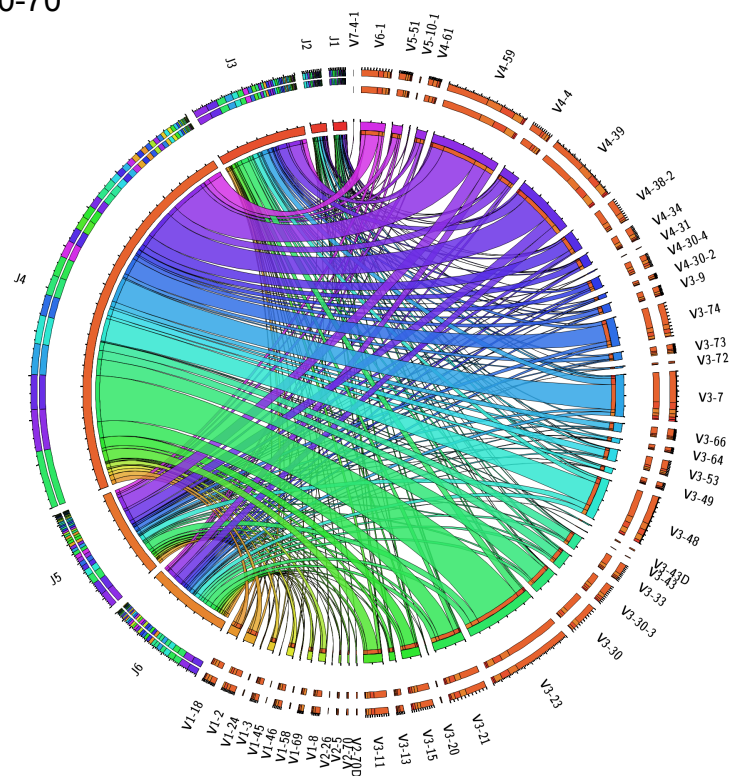

B

non-switched memory

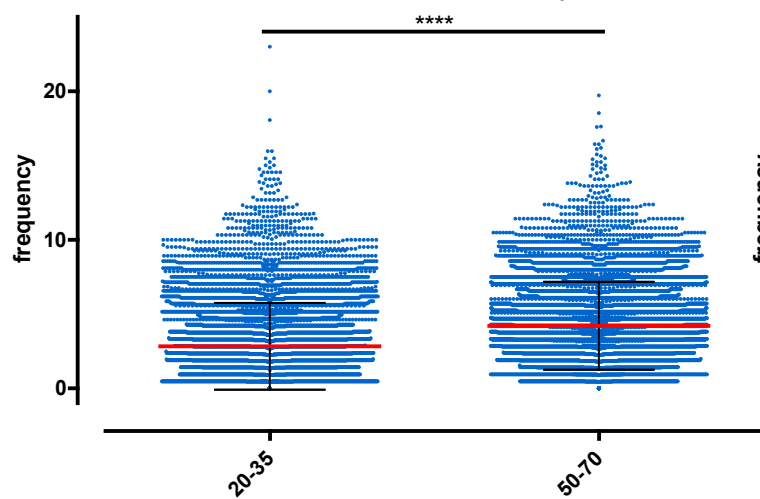

C

IgM-only

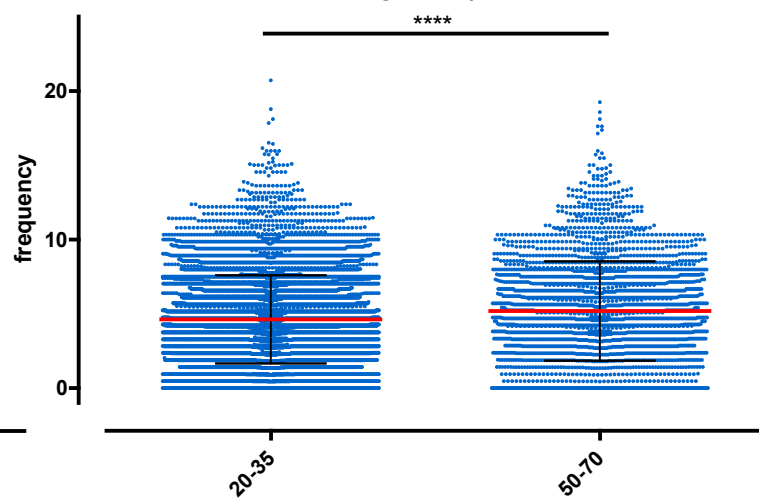

D

CD27+IgG+ switched memory

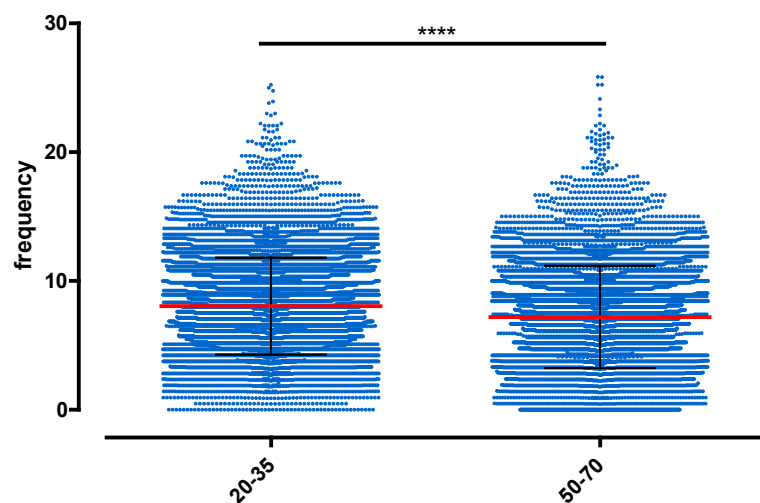

Supplement: Supplementary file 5 — Figure S5. Minor differences in BCR repertoire of antigen-experienced B cell subpopulations in different age groups. (PDF 8560 kb) [file 12979_2019_163_MOESM5_ESM.pdf]
