# Supplementary material for: The presence of CLL-associated stereotypic B cell receptors in the normal BCR repertoire from healthy individuals increases with age
Source: Immun Ageing. 2019 Aug 28;16:22. doi: 10.1186/s12979-019-0163-x (PMC6714092; doi:10.1186/s12979-019-0163-x)
Supplement: Supplementary file 6 — Table S1. Definition of human B cell subpopulations. Table S2 Overview of productive and unique IGH sequences in NGS analysis. Table S3 Composition of different antibody panels for staining B cell subpopulations. (DOCX 26 kb) [file 12979_2019_163_MOESM6_ESM.docx]

**SUPPLEMENTARY INFORMATION TO**

**The presence of CLL-associated stereotypic B cell receptors in the normal BCR repertoire from healthy individuals increases with age**

*Alice F. Muggen^1^. Madelon de Jong^1^. Ingrid L.M. Wolvers-Tettero^1^. Martine J. Kallemeijn^1^. Cristina Teodósio^1.‡^. Nikos Darzentas^2.3^. Ralph Stadhouders^4^. Hanna IJspeert^1 †^. Mirjam van de Burg^1 †^. Wilfred van IJcken^5^. Jan A.N. Verhaar^6^. Wayel H. Abdulahad^7^. Elisabeth Brouwer^7^. Annemieke M. H. Boots^7^. Rudi W. Hendriks^4^. Jacques J.M. van Dongen^1. ‡^. Anton W. Langerak^1^*

*^1^Department of Immunology.* *Laboratory Medical Immunology. Erasmus MC. Rotterdam. The Netherlands. ^2^Central European Institute of Technology. Masaryk University. Brno. Czech Republic. ^3^Department of Internal Medicine. University Schleswig-Holstein. Kiel. Germany. ^4^Department of Pulmonary Medicine. Erasmus MC. Rotterdam. The Netherlands. ^5^Biomics Core Facility. Erasmus MC. Rotterdam. The Netherlands. ^6^Department of Orthopedics. Erasmus MC. Rotterdam. The Netherlands. ^7^Department of Rheumatology and Clinical Immunology. University Medical Center Groningen. Groningen. The Netherlands*

^†^ Present address: Department of Pediatrics. Leiden University Medical Center. Leiden. The Netherlands

^‡^ Present address: Department of Immunohematology and Blood Transfusion. Leiden University Medical Center. Leiden. The Netherlands

**SUPPLEMENTARY TABLES**

**Table S1 Definition of human B cell subpopulations**

| **B cell subpopulation** | **Markers** |
| --- | --- |
| transitional B cells | CD19+/CD38hi/CD27- |
| naive mature B cells | CD19+/CD38-/CD27-/IgM+/IgD+ |
| non-switched memory B cells | CD19+/CD38-/CD27+/IgM+/IgD+ |
| IgM-only B cells | CD19+/CD38-/CD27+/IgM+/IgD- |
| CD27+ or – IgG+ switched memory B cells | CD19+/CD38-/CD27+ or -/IgM-/IgD-/IgG+ |
| CD27+ or – IgA+ switched memory B cells | CD19+/CD38-/CD27+ or -/IgM-/IgD-/IgA+ |
| CD27+ or – IgE+ switched memory B cells | CD19+/CD38-/CD27+ or -/IgM-/IgD-/IgE+ |
| plasma blasts | CD19+/CD38^hi^/CD27+ |
| CD5+/CD43+ B cells | CD19+/CD38-/CD5+/CD43+ |
| CD21^low^ B cells | CD19+/CD38^dim^/CD21^low^ |

**Table S2 Overview of productive and unique IGH sequences in NGS analysis**

| **B cell subpopulation** | **Age group** | **Productive sequences** | **Unique sequences** |
| --- | --- | --- | --- |
| naïve mature B cells | <50 (n=5) | 20507 | 14257 |
|  | 50-70 (n=5) | 31211 | 20367 |
|  | >70 (n=4) | 657 | 648 |
| non-switched memory B cells | <50 (n=5) | 17793 | 9495 |
|  | 50-70 (n=4) | 16012 | 10973 |
| IgM-only B cells | <50 (n=5) | 26519 | 10112 |
|  | 50-70 (n=3) | 7248 | 3200 |
| switched memory B cells (CD27+IgG+) | <50 (n=5) | 27606 | 9329 |
|  | 50-70 (n=4) | 15030 | 8994 |

**Table S3 Composition of different antibody panels for staining B cell subpopulations**

| **Panel 1** |  | **Panel 2** |  |
| --- | --- | --- | --- |
| Marker | Fluorochrome | Marker | Fluorochrome |
| **CD27** | BV421 | **CD27** | BV421 |
| **IgM** | BV510 | **IgM** | BV510 |
| **CD20** | BV605 | **CD20** | BV605 |
| **CD21** | BV711 | **CD21** | BV711 |
| IgE | FITC | CD43 | FITC |
| IgA | FITC |  |  |
| IgG | PE | CD11b | PE |
| IgA | PE |  |  |
| **IgD** | PE-CF594 | **IgD** | PE-CF594 |
| CD79b | PerCP | CD11c | PerCP |
| **CD19** | PE-Cy7 | **CD19** | PE-Cy7 |
| CD23 | APC | CD5 | APC |
| **CD38** | APC-H7 | **CD38** | APC-H7 |

*To allow combining data from the two panels. a backbone of markers (indicated in bold) was used.*
