# Supplementary material for: The presence of CLL-associated stereotypic B cell receptors in the normal BCR repertoire from healthy individuals increases with age
Source: Immun Ageing. 2019 Aug 28;16:22. doi: 10.1186/s12979-019-0163-x (PMC6714092; doi:10.1186/s12979-019-0163-x)
Supplement: Supplementary file 7 — Cross-validation of IGH assay between different NGS platforms. (DOCX 32 kb) [file 12979_2019_163_MOESM7_ESM.docx]

**SUPPLEMENTARY INFORMATION**

Cross-validation between different NGS platforms was performed using DNA from 8 controls that were used for paired analysis of IGH DNA and RNA/cDNA libraries on 454 (Roche. Branford. CT) and MiSeq (Illumina. San Diego. CA) platforms and data analysis using the ImmunoGlobulin galaxy (IGGalaxy) pipeline (Moorhouse et al.. 2014).

**Comparison of productive and unique productive sequences**

| Donor |  | All | Productive |  | Unique Productive |  |
| --- | --- | --- | --- | --- | --- | --- |
| NWK16 | 454 | 4363 | 2849 | 65% | 1539 | 35% |
| NWK214 | 454 | 7640 | 3845 | 50% | 1951 | 26% |
| NWK276 | 454 | 8183 | 6486 | 79% | 4286 | 52% |
| NWK279 | 454 | 11111 | 8517 | 77% | 4716 | 42% |
| NWK31 | 454 | 3322 | 1226 | 37% | 928 | 28% |
| NWK43 | 454 | 7868 | 4994 | 63% | 2669 | 34% |
| NWK53 | 454 | 32403 | 24107 | 74% | 3449 | 11% |
| NWK54 | 454 | 4547 | 2966 | 65% | 1528 | 34% |
| Mean | 454 |  |  | 64% |  | 33% |
| NWK16 | ILLUMINA | 29043 | 21964 | 76% | 9858 | 34% |
| NWK214 | ILLUMINA | 30202 | 20884 | 69% | 10790 | 36% |
| NWK276 | ILLUMINA | 41469 | 28165 | 68% | 16173 | 39% |
| NWK279 | ILLUMINA | 36737 | 24792 | 67% | 13725 | 37% |
| NWK31 | ILLUMINA | 44931 | 33886 | 75% | 19209 | 43% |
| NWK43 | ILLUMINA | 41800 | 19032 | 46% | 13834 | 33% |
| NWK53 | ILLUMINA | 44482 | 35421 | 80% | 11212 | 25% |
| NWK54 | ILLUMINA | 30746 | 19682 | 64% | 9498 | 31% |
| Mean | ILLUMINA |  |  | 68% |  | 35% |

**Comparison of junctional region characteristics**

| Donor |  | Total,Del | Total,N | Total,P | CDR3,Length |
| --- | --- | --- | --- | --- | --- |
| NWK16 | 454 | 18.9 | 11 | 0.6 | 15 |
| NWK214 | 454 | 20.2 | 11.9 | 0.5 | 14 |
| NWK276 | 454 | 17.2 | 12.5 | 0.6 | 16 |
| NWK279 | 454 | 17.7 | 12.8 | 0.5 | 16 |
| NWK31 | 454 | 19.8 | 10.9 | 0.5 | 14 |
| NWK43 | 454 | 21.1 | 11 | 0.4 | 14 |
| NWK53 | 454 | 20.8 | 12.1 | 0.5 | 14 |
| NWK54 | 454 | 18.7 | 11.4 | 0.4 | 14 |
| Mean | 454 | 18.8 | 11.2 | 0.5 | 14.5 |
| NWK16 | ILLUMINA | 19.5 | 12 | 0.5 | 15 |
| NWK214 | ILLUMINA | 20.9 | 12.4 | 0.4 | 14 |
| NWK276 | ILLUMINA | 17.4 | 11.7 | 0.6 | 16 |
| NWK279 | ILLUMINA | 17.7 | 12.3 | 0.6 | 16 |
| NWK31 | ILLUMINA | 18.9 | 11.7 | 0.5 | 15 |
| NWK43 | ILLUMINA | 19.7 | 11 | 0.5 | 14 |
| NWK53 | ILLUMINA | 21.5 | 12.8 | 0.4 | 14 |
| NWK54 | ILLUMINA | 19 | 12.5 | 0.4 | 14 |
| Mean | ILLUMINA | 19.25 | 12.25 | 0.45 | 14.5 |

**Comparison of V subgroup usage**

| Donor |  | IGHV1 | IGHV2 | IGHV3 | IGHV4 | IGHV5 | IGHV6 | IGHV7 |
| --- | --- | --- | --- | --- | --- | --- | --- | --- |
| NWK16 | 454 | 8.77 | 0.06 | 65.95 | 19.75 | 5.26 | 0.19 |  |
| NWK214 | 454 | 8.25 | 0.46 | 40.80 | 47.87 | 1.90 | 0.72 |  |
| NWK276 | 454 | 7.44 | 1.61 | 55.27 | 32.13 | 2.24 | 1.31 |  |
| NWK279 | 454 | 8.69 | 1.38 | 54.52 | 32.46 | 2.04 | 0.91 |  |
| NWK31 | 454 | 14.44 | 1.62 | 51.08 | 30.39 | 1.94 | 0.54 |  |
| NWK43 | 454 | 4.57 | 1.09 | 61.22 | 26.71 | 3.11 | 3.18 | 0.11 |
| NWK53 | 454 | 7.36 | 0.58 | 66.98 | 20.50 | 3.89 | 0.70 |  |
| NWK54 | 454 | 3.93 | 0.20 | 69.31 | 21.34 | 4.65 | 0.46 | 0.13 |
| Mean | 454 | 7.93 | 0.87 | 58.14 | 28.89 | 3.13 | 1.00 | 0.12 |
| NWK16 | ILLUMINA | 19.32 | 0.87 | 53.04 | 22.95 | 3.62 | 0.19 |  |
| NWK214 | ILLUMINA | 0.38 | 0.05 | 66.29 | 30.92 | 1.42 | 0.94 | 0.01 |
| NWK276 | ILLUMINA | 39.49 | 1.79 | 40.75 | 8.64 | 8.68 | 0.64 | 0.01 |
| NWK279 | ILLUMINA | 44.92 | 1.70 | 39.81 | 6.87 | 5.68 | 0.98 | 0.03 |
| NWK31 | ILLUMINA | 15.20 | 2.34 | 40.05 | 26.12 | 14.35 | 1.38 | 0.56 |
| NWK43 | ILLUMINA | 10.16 | 2.11 | 36.29 | 35.39 | 12.06 | 3.43 | 0.56 |
| NWK53 | ILLUMINA | 16.33 | 1.31 | 40.90 | 36.59 | 4.17 | 0.67 | 0.02 |
| NWK54 | ILLUMINA | 0.64 | 0.03 | 48.96 | 46.11 | 3.33 | 0.93 |  |
| Mean | ILLUMINA | 18.30 | 1.28 | 45.76 | 26.70 | 6.66 | 1.14 | 0.20 |

**Comparison of J gene usage**

| \| Donor \|  \| IGHJ1 \| IGHJ2 \| IGHJ3 \| IGHJ4 \| IGHJ5 \| IGHJ6 \| \| --- \| --- \| --- \| --- \| --- \| --- \| --- \| --- \| \| NWK16 \| 454 \| 1.30 \| 2.86 \| 17.48 \| 45.35 \| 8.77 \| 24.24 \| \| NWK214 \| 454 \| 2.72 \| 3.74 \| 9.33 \| 52.49 \| 15.89 \| 15.84 \| \| NWK276 \| 454 \| 1.33 \| 2.75 \| 11.78 \| 43.89 \| 11.06 \| 29.19 \| \| NWK279 \| 454 \| 1.95 \| 2.84 \| 12.77 \| 43.83 \| 12.13 \| 26.48 \| \| NWK31 \| 454 \| 5.50 \| 4.42 \| 8.19 \| 48.38 \| 14.87 \| 18.64 \| \| NWK43 \| 454 \| 3.33 \| 4.50 \| 12.51 \| 50.88 \| 12.89 \| 15.89 \| \| NWK53 \| 454 \| 2.17 \| 4.03 \| 9.51 \| 53.93 \| 13.31 \| 17.05 \| \| NWK54 \| 454 \| 2.29 \| 2.88 \| 12.30 \| 54.25 \| 11.26 \| 17.02 \| \| Mean \| 454 \| 2.57 \| 3.50 \| 11.73 \| 49.13 \| 12.52 \| 20.54 \| \| NWK16 \| ILLUMINA \| 1.13 \| 3.95 \| 15.56 \| 41.72 \| 11.09 \| 26.56 \| \| NWK214 \| ILLUMINA \| 2.80 \| 4.08 \| 9.84 \| 53.20 \| 14.09 \| 16.00 \| \| NWK276 \| ILLUMINA \| 1.16 \| 1.28 \| 10.33 \| 37.28 \| 11.21 \| 38.75 \| \| NWK279 \| ILLUMINA \| 1.88 \| 1.54 \| 14.70 \| 35.72 \| 10.14 \| 36.02 \| \| NWK31 \| ILLUMINA \| 2.76 \| 3.00 \| 12.04 \| 49.73 \| 13.00 \| 19.46 \| \| NWK43 \| ILLUMINA \| 2.76 \| 3.79 \| 12.28 \| 51.63 \| 14.03 \| 15.50 \| \| NWK53 \| ILLUMINA \| 1.96 \| 3.29 \| 10.60 \| 49.31 \| 15.61 \| 19.23 \| \| NWK54 \| ILLUMINA \| 2.10 \| 2.07 \| 12.16 \| 54.32 \| 13.22 \| 16.13 \| \| Mean \| ILLUMINA \| 2.07 \| 2.88 \| 12.19 \| 46.61 \| 12.80 \| 23.46 \| |  |  |  |  |  |  |  |
| --- | --- | --- | --- | --- | --- | --- | --- | --- | --- | --- | --- | --- | --- | --- | --- | --- | --- | --- | --- | --- | --- | --- | --- | --- | --- | --- | --- | --- | --- | --- | --- | --- | --- | --- | --- | --- | --- | --- | --- | --- | --- | --- | --- | --- | --- | --- | --- | --- | --- | --- | --- | --- | --- | --- | --- | --- | --- | --- | --- | --- | --- | --- | --- | --- | --- | --- | --- | --- | --- | --- | --- | --- | --- | --- | --- | --- | --- | --- | --- | --- | --- | --- | --- | --- | --- | --- | --- | --- | --- | --- | --- | --- | --- | --- | --- | --- | --- | --- | --- | --- | --- | --- | --- | --- | --- | --- | --- | --- | --- | --- | --- | --- | --- | --- | --- | --- | --- | --- | --- | --- | --- | --- | --- | --- | --- | --- | --- | --- | --- | --- | --- | --- | --- | --- | --- | --- | --- | --- | --- | --- | --- | --- | --- | --- | --- | --- | --- | --- | --- | --- | --- | --- | --- | --- | --- | --- | --- | --- | --- |
